# Cross-tissue transcriptome-wide association and Mendelian randomization identify RALB as a susceptibility gene for breast hypertrophy

**DOI:** 10.1097/MD.0000000000045872

**Published:** 2025-11-14

**Authors:** Junwei Gu, Jingshuang Chen, Zujian Hu, Hua Luo

**Affiliations:** a Department of Breast Surgery, Hangzhou TCM Hospital Affiliated to Zhejiang Chinese Medical University, Hangzhou Hospital of Traditional Chinese Medicine, Hangzhou, Zhejiang, China; b The Second Affiliated Hospital of Zhejiang Chinese Medical University, Reproductive Medicine Center, Hangzhou, China.

**Keywords:** breast hypertrophy, eQTL, FUSION, genetic susceptibility, GWAS, RALB, TWAS, UTMOST

## Abstract

Breast hypertrophy is a pathological condition characterized by abnormal enlargement of the breasts due to excessive glandular and adipose tissue proliferation. It is associated with physical discomfort and reduced quality of life. However, its underlying genetic basis remains largely unexplored. We conducted a cross-tissue transcriptome-wide association study (TWAS) using the UTMOST framework, integrating expression quantitative trait locus data from GTEx and GWAS summary statistics from the FinnGen database (7272 cases, 258,508 controls). Additional validation was performed using single-tissue TWAS (FUSION), gene-level association testing (Multi-marker Analysis of GenoMic Annotation (MAGMA)), fine-mapping (FOCUS), and conditional and joint analyses. Key candidate genes were subjected to Mendelian randomization (MR) and Bayesian colocalization analyses to investigate causality. A total of 44 genes were significantly associated with breast hypertrophy in cross-tissue TWAS (false discovery rate < 0.05), and 4 were replicated in whole blood via FUSION. Fine-mapping identified RAS Like Proto-Oncogene B (RALB) as the most probable causal gene (PIP = 0.98), supported by MAGMA, conditional analyses, and colocalization (PPH4 = 0.922). Mendelian randomization confirmed a significant causal relationship between elevated RALB expression and increased risk of breast hypertrophy (odds ratio = 1.26; 95% confidence interval: 1.17–1.35; *P* <  .001). Our integrative genomic analyses identified RALB as a robust susceptibility gene for breast hypertrophy. These findings provide novel insights into the genetic etiology of breast hypertrophy and may facilitate future development of molecular diagnostics or targeted therapies.

## 1. Introduction

Breast hypertrophy, is a pathological condition characterized by excessive proliferation of mammary glandular and adipose tissues, leading to abnormal breast enlargement.^[1]^ Diagnostic criteria typically define it as unilateral breast volume exceeding physiological limits (>400 mL for moderate hypertrophy, >1000 mL for severe hypertrophy, and >1500 mL for macromastia) or breast weight surpassing 3% of total body weight.^[2]^ The morphological hallmark of this condition includes altered breast contour, presenting as a pendulous or “teardrop-shaped” silhouette with downward displacement of the nipple-areolar complex, alongside cutaneous venous prominence and hyperpigmentation. Histopathological analysis further reveals marked hyperplasia of glandular, adipose, and fibrous tissues, with microscopic findings of gray-red glandular proliferation interlaced with yellowish adipose tissue.^[1–3]^ These structural abnormalities correlate closely with physical symptoms such as breast weight-induced shoulder/back pain, frictional dermatitis, and restricted mobility, which are exacerbated in severe cases by chronic gravitational strain leading to spinal kyphosis.^[2,4,5]^ Beyond anatomical disruptions, the psychosocial impact of breast hypertrophy manifests as body image disturbances, anxiety, social withdrawal, and diminished quality of life, underscoring the multisystem burden of this condition.^[5,6]^

Transcriptome-wide association studies (TWASs) integrate expression quantitative trait locus (eQTL) data with genome-wide association study (GWAS) summary statistics to identify candidate genes and elucidate gene-disease associations.^[7]^ Compared to traditional GWAS, TWAS enables the prediction of gene expression effects on phenotypes, offering deeper insights into the biology of complex traits.^[8,9]^ Unified Test for Molecular Signatures (UTMOST), a representative cross-tissue TWAS method, captures both shared eQTL effects across tissues and tissue-specific regulatory features,^[10]^ making it particularly suitable for multisystem disease research. In recent years, such approaches have been widely applied to psychiatric, inflammatory, and cancer-related disorders, facilitating the discovery of novel susceptibility genes and revealing the role of cross-tissue genetic effects in disease mechanisms.^[11–13]^

In this study, we employed the UTMOST approach to systematically integrate eQTLs data from the genotype-tissue expression (GTEx) project with GWAS data for patients with breast hypertrophy from the Finnish biobank and registry-based genomic study (FINNGEN) database, conducting a cross-tissue TWAS. In parallel, we applied 3 computational methods – FUSION, fine-mapping of causal gene sets (FOCUS), and Multi-marker Analysis of GenoMic Annotation (MAGMA) – for multiple validation of the identified candidate susceptibility genes. For genes showing independent genetic associations with the breast hypertrophy phenotype, we further performed conditional and joint analyses. Building upon these results, we conducted rigorous Mendelian randomization and colocalization analyses to explore the causal relationships and underlying genetic mechanisms linking these genes to the breast hypertrophy phenotype.

## 2. Material and methods

### 2.1. Data source of breast hypertrophy and study design

**Figure 1.**
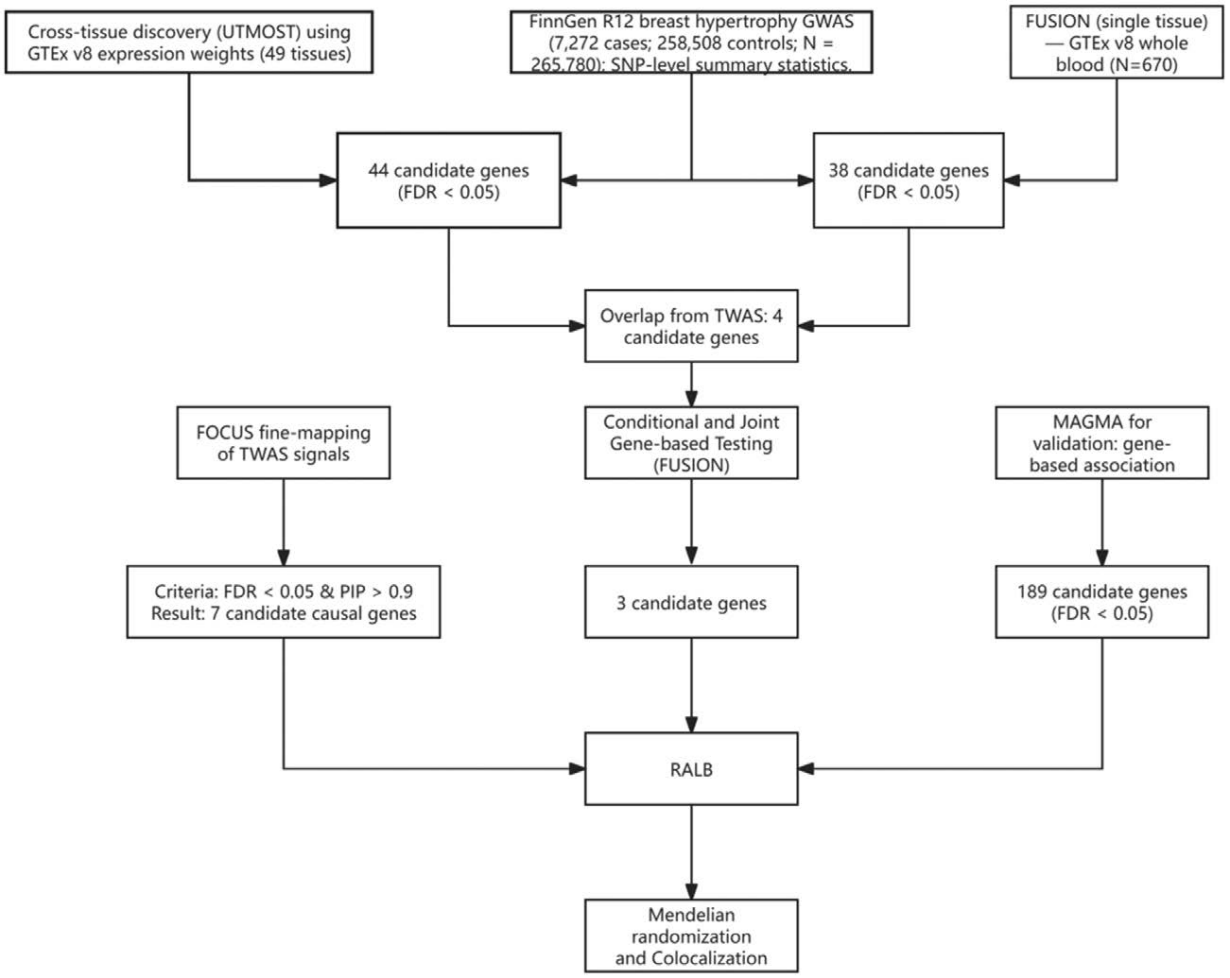
Study workflow for integrative genetics of breast hypertrophy (FinnGen R12). FinnGen = Finnish biobank and registry-based genomic study.

The FinnGen project integrates genotype data from Finnish biobanks with national health registry information to conduct phenotype-disease association analyses based on the International Classification of Diseases, 10th Revision classification system. This study utilized summary statistics from the R12 release of the FinnGen database, including 7272 cases of breast hypertrophy and 258,508 controls. (Download link: https://storage.googleapis.com/finngen-public-data-r12/summary_stats/release/finngen_R12_N14_HYPERTROPHYBREAST.gz) An overview of the end-to-end analytic workflow is shown in Figure 1. This study used de-identified, publicly available summary data from FinnGen and GTEx; per journal policy, no additional IRB approval or informed consent was required.

### 2.2. UTMOST analysis

In this study, we employed the UTMOST to perform cross-tissue transcriptome-wide association studies (TWAS). UTMOST integrates gene-trait associations across multiple tissues, enhancing the power to identify genes related to complex traits, while addressing limitations caused by small sample sizes in individual tissues.^[10]^ Using gene expression data from 49 tissues in the GTEx project and GWAS summary statistics, we applied UTMOST to construct single nucleotide polymorphism (SNP) effect models across linkage disequilibrium (LD) regions for single-tissue gene-trait association analyses. The results were then combined using the Generalized Berk–Jones test, which accounts for the covariance structure among tissues to improve cross-tissue inference accuracy.^[14]^ Statistical significance was determined using false discovery rate (FDR) correction, with FDR < 0.05 considered significant. The UTMOST tool and documentation are available at: https://github.com/Joker-Jerome/UTMOST.

### 2.3. FUSION analysis

We performed single-tissue transcriptome-wide association studies (TWAS) for breast hypertrophy using the FUSION method (https://github.com/gusevlab/fusion_twas), integrating GWAS summary statistics for breast hypertrophy with eQTLs data from 49 human tissues provided by GTEx v8.^[7]^ FUSION builds gene expression prediction models based on reference samples with both genotype and expression data, selecting cis-SNPs within ±1 Mb of each gene. Multiple statistical models, including BLUP, BSLMM, LASSO, Elastic Net, and Top1, were used to estimate the contribution of SNPs to gene expression. LD structure was estimated using an EUR reference panel from the 1000 Genomes Project. TWAS statistics were then calculated by combining GWAS *Z*-scores with predicted gene expression weights. Multiple testing correction was performed using the FDR, with statistical significance defined as FDR < 0.05.

### 2.4. Conditional and joint gene-based testing (FUSION)

Following single-tissue TWAS, we applied the FUSION assoc_test (conditional) and joint_test (joint) procedures to evaluate the independence of gene-level TWAS signals within each locus while accounting for local LD.^[7,15]^ LD was estimated using an EUR reference panel from the 1000 Genomes Project. Genes that remained significant in the joint model were considered independently associated, whereas signals that attenuated after conditioning were regarded as marginal/LD-dependent. This locus-level dissection reduces inflation from correlated predicted expression and improves the interpretability of TWAS associations.

### 2.5. MAGMA analysis

In this study, we employed MAGMA software (v1.08) to perform genes and gene set-based association analyses of the GWAS results. Using default parameters, MAGMA aggregates SNP-level association statistics to compute gene-level scores, enabling the evaluation of gene–phenotype associations and the identification of functional genes influenced by multiple small-effect SNPs.^[16,17]^ Detailed methodology is available in the official MAGMA documentation (https://cncr.nl/research/magma/).^[18]^

### 2.6. FOCUS analysis

We applied FOCUS to perform probabilistic fine-mapping of TWAS results, aiming to identify candidate genes with evidence of causality. FOCUS integrates GWAS summary statistics and eQTL-derived expression weights to model the correlation structure among TWAS signals and assigns each gene a posterior inclusion probability (PIP) reflecting its likelihood of explaining the association within a given risk locus.^[19]^ A precomputed weight reference panel from multiple tissues was used. Genes with PIP ≥ 0.9 and GWAS *P* < 5 × 10^−8^ were considered as likely causal.

### 2.7. Integration of multi-method results and Mendelian randomization (MR) analysis

We integrated the risk genes identified by 4 analytical approaches – MAGMA, UTMOST, FUSION, and FOCUS – and selected their intersection as key candidate genes for subsequent Mendelian randomization (MR) and Bayesian colocalization analyses.

MR evaluates the causal effect of an exposure on an outcome using genetic variants significantly associated with the exposure as instrumental variables (IVs).^[20]^ Valid IVs were required to meet the following criteria: strong association with the exposure (*P* < 5 × 10^−8^), no association with confounders, and an effect on the outcome only through the exposure. IVs were clumped within a 10,000 kb window using an LD threshold of *r*^2^ < 0.1 and harmonized across exposure and outcome GWAS datasets. When only one IV was available, the Wald ratio method was used; with 2 or more IVs, inverse-variance weighted analysis was applied.^[21]^ Statistical significance was defined as *P* < .05. Sensitivity analyses – including Mendelian Randomization Egger Regression (MR-Egger), weighted median, and others – were subsequently performed, along with assessments for horizontal pleiotropy (MR-Egger intercept), heterogeneity (Cochran *Q*), and outliers (Mendelian Randomization Pleiotropy RESidual Sum and Outlier). All analyses were conducted using the TwoSampleMR R package.

### 2.8. Bayesian colocalization analysis

To assess whether GWAS and eQTL signals share a common causal variant, we performed Bayesian colocalization analysis using the R package coloc (v5.2.3) with default parameters.^[22]^ The method estimates the posterior probability of hypothesis (PPH) for 5 hypotheses: H0, neither trait is associated; H1, associated with trait 1 only; H2, associated with trait 2 only; H3, both traits are associated but driven by distinct causal variants; and H4, both traits are associated and share a common causal variant. Based on previous studies, PPH4 > 0.8 indicates strong evidence for colocalization, while PPH4 > 0.5 suggests moderate colocalization. The coloc package is available at: https://github.com/chr1swallace/coloc.

## 3. Results

### 3.1. Cross-tissue and single-tissue TWAS analysis

**Figure 2.**
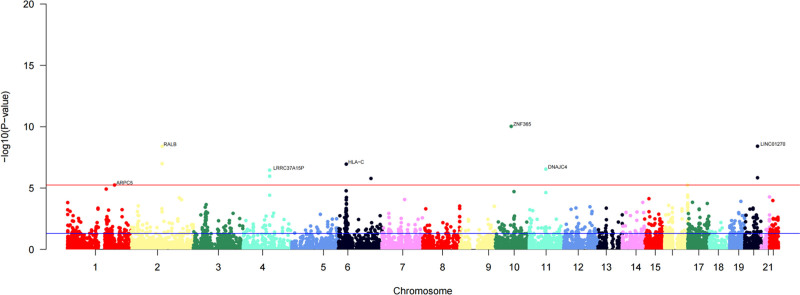
Manhattan plot of FUSION analysis results. FUSION = functional summary-based imputation.

Cross-tissue TWAS analysis identified a total of 338 genes showing statistically significant associations (*P* < .05, Table S1, Supplemental Digital Content, https://links.lww.com/MD/Q639), among which 44 genes remained significant after stringent FDR correction (FDR < 0.05, Table 1). These genes may play key roles in the pathogenesis of breast hypertrophy and provide potential targets for subsequent functional studies. To enhance the rigor and reliability of our findings, we further performed single-tissue TWAS validation analysis. Using the FUSION method, we analyzed genotype and expression data from whole blood tissue in the GTEx dataset. This analysis identified 38 genes that retained significant TWAS signals after FDR correction (FDR < 0.05, Table 2 and Fig. 2). Overall, by applying stringent thresholds in both cross-tissue and single-tissue analyses, we identified 4 overlapping candidate genes, further supporting their potential roles in the development of breast hypertrophy.

**Table 1:** The significant genes for breast hypertrophy risk in cross-tissue TWAS analysis.

Gene	chr	test_score	*P*_value	FDR
MRPS18C	4	23.94	1.84e−11	6.89e−08
NKX6-1	4	18	9.21e−09	1.72e−05
WDFY3	4	15.19	2.71e−07	3.38e−04
CENPE	4	13.57	4.04e−07	3.39e−04
LRRC37A15P	4	13.06	4.53e−07	3.39e−04
KRT8P46	4	12.72	7.67e−07	4.78e−04
TACR3	4	13.13	1.95e−06	1.04e−03
UBE2D3	4	11.51	3.54e−06	1.65e−03
CISD2	4	10.43	6.41e−06	2.66e−03
DBI	2	11.44	9.05e−06	3.39e−03
RP11-10L12.4	4	9.71	1.56e−05	5.29e−03
TNS1	2	10.77	2.02e−05	6.29e−03
PTRHD1	2	10.73	2.46e−05	7.08e−03
RALB	2	9.8	3.05e−05	8.14e−03
CALCRL	2	9.78	4.18e−05	9.77e−03
TNP1	2	10	4.10e−05	9.77e−03
BDH2	4	8.21	5.30e−05	1.02e−02
ARL9	4	10.39	5.44e−05	1.02e−02
AC092484.1	2	8.72	4.97e−05	1.02e−02
TMEM185B	2	8.02	5.06e−05	1.02e−02
COBLL1	2	9.08	7.63e−05	1.33e−02
NTSR2	2	9.3	7.80e−05	1.33e−02
AC013472.4	2	9.79	8.34e−05	1.36e−02
ZC3H15	2	9.42	8.84e−05	1.37e−02
SELENOI	2	9.16	9.30e−05	1.37e−02
SLC9B2	4	9.42	9.54e−05	1.37e−02
MTND5P28	2	8.42	1.11e−04	1.54e−02
SDC4P	22	8.2	1.16e−04	1.55e−02
IGKJ2	2	8.62	1.28e−04	1.65e−02
TMEM177	2	8.62	1.34e−04	1.67e−02
CDS1	4	9.04	1.42e−04	1.71e−02
CRYBB2P1	22	7.86	1.70e−04	1.99e−02
AC007319.1	2	7.81	1.82e−04	2.01e−02
AC007238.1	2	8.47	1.78e−04	2.01e−02
GRK3	22	7.55	2.15e−04	2.30e−02
SLC39A8	4	8.02	2.21e−04	2.30e−02
RP11−94B19.5	18	3.68	2.32e−04	2.35e−02
NYAP2	2	8	3.00e−04	2.87e−02
ARHGAP25	2	7.55	2.99e−04	2.87e−02
TLR10	4	9.28	3.06e−04	2.87e−02
MTCO3P43	2	7.2	3.21e−04	2.93e−02
PTPN4	2	7.48	3.91e−04	3.43e−02
AC016717.1	2	7.67	3.94e−04	3.43e−02
EFEMP1	2	7.39	4.64e−04	3.95e−02

CALCRL = calcitonin receptor-like receptor, FDR = false discovery rate, KRT8P46 = keratin 8 pseudogene 46, LRRC37A15P = leucine rich repeat containing 37 member A15 pseudogene, TWAS = transcriptome-wide association study.

**Table 2:** The significant breast hypertrophy risk genes identified by FUSION.

GENE	CHR	TWAS.Z	TWAS.P	FDR
ZNF365	10	6.48	9.35e−11	8.19e−07
RALB	2	5.89	3.95e−09	1.15e−05
LINC01270	20	−5.89	3.89e−09	1.15e−05
INHBB	2	5.32	1.02e−07	1.96e−04
HLA-C	6	−5.31	1.12e−07	1.96e−04
DNAJC4	11	−5.13	2.86e−07	4.17e−04
LRRC37A15P	4	5.1	3.47e−07	4.34e−04
KRT8P46	4	4.87	1.09e−06	1.06e−03
RP11-10L12.2	4	4.87	1.09e−06	1.06e−03
LINC01271	20	−4.82	1.45e−06	1.27e−03
KIAA0408	6	−4.79	1.66e−06	1.32e−03
ARPC5	1	−4.54	5.67e−06	3.95e−03
RP11-46C24.7	16	−4.53	5.86e−06	3.95e−03
SEMA6C	1	4.38	1.21e−05	7.57e−03
LINC00243	6	−4.3	1.68e−05	9.81e−03
DNAJC9-AS1	10	4.27	1.95e−05	1.07e−02
TRPT1	11	4.23	2.34e−05	1.21e−02
ANKRD11	16	4.12	3.81e−05	1.78e−02
RP11-10L12.1	4	4.12	3.86e−05	1.78e−02
DSTNP1	21	−4.04	5.28e−05	2.31e−02
CALCRL	2	4	6.34e−05	2.31e−02
SPIRE2	16	−4.03	5.63e−05	2.31e−02
ATP6V1G2	6	4.01	6.19e−05	2.31e−02
C4B	6	−4.02	5.85e−05	2.31e−02
BAHD1	15	3.96	7.36e−05	2.58e−02
STK17B	2	−3.92	8.91e−05	2.89e−02
STEAP4	7	−3.93	8.64e−05	2.89e−02
CCHCR1	6	−3.91	9.32e−05	2.91e−02
THOC5	22	−3.88	1.04e−04	3.14e−02
KCNN4	19	−3.84	1.22e−04	3.56e−02
ALKBH5	17	−3.8	1.45e−04	4.02e−02
WDR25	14	−3.8	1.47e−04	4.02e−02
PERM1	1	3.79	1.53e−04	4.06e−02
SPG7	16	3.76	1.68e−04	4.33e−02
TRIM47	17	3.74	1.81e−04	4.52e−02
NEU1	6	3.74	1.86e−04	4.52e−02
C2	6	−3.71	2.06e−04	4.87e−02
RP11-104N10.2	16	3.7	2.14e−04	4.93e−02

CALCRL = calcitonin receptor-like receptor, FDR = false discovery rate, FUSION = functional summary-based imputation, KRT8P46 = keratin 8 pseudogene 46, LRRC37A15P = leucine rich repeat containing 37 member a15 pseudogene, TWAS = transcriptome-wide association study.

### 3.2. Conditional and joint gene-based analyses (FUSION)

**Figure 3.**
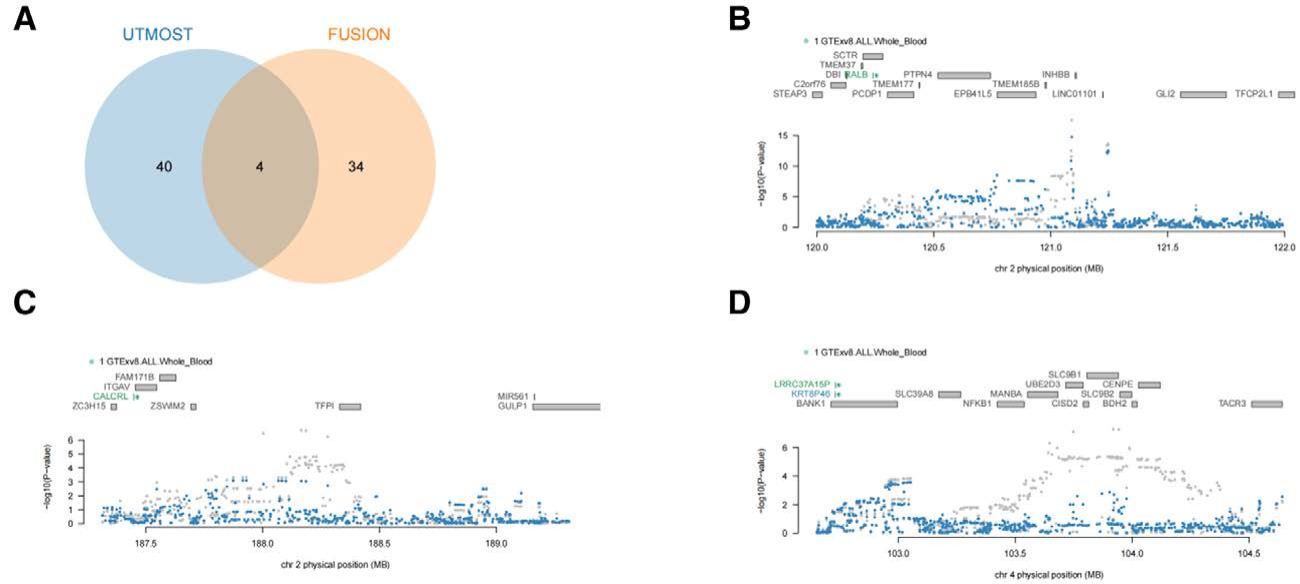
Conditional and joint gene-based TWAS analyses (FUSION). (A) Venn diagram showing the overlap of significant genes from cross-tissue UTMOST and single-tissue FUSION analyses (FDR < 0.05). (B–D) Locus panels for 2q14.3 (B), 2q32.1 (C), and 4q24 (D) based on FUSION joint modeling. The x-axis shows genomic position (hg19; Mb) and the y-axis shows TWAS − log10(*P*). Genes highlighted in green remained significant in the joint model (independent signals), whereas blue genes attenuated after conditioning (marginal/LD-dependent). FDR = false discovery rate, FUSION = functional summary-based imputation, LD = linkage disequilibrium, TWAS = transcriptome-wide association study, UTMOST = unified test for molecular signatures.

As shown in Figure 3A, applying FDR-controlled thresholds (FDR < 0.05) in both cross-tissue (UTMOST) and single-tissue (FUSION) TWAS yielded 4 overlapping candidate genes. To assess whether these signals were independent, we performed gene-level conditional and joint testing in FUSION (“assoc_test”/“joint_test”). The results indicated that 3 loci – 2q14.3 (RAS Like Proto-Oncogene B (RALB), Fig. 3B), 2q32.1 (calcitonin receptor-like receptor (CALCRL), Fig. 3C), and 4q24 (Leucine Rich Repeat Containing 37 Member A15 Pseudogene (LRRC37A15P), Fig. 3D) – harbor multiple independent gene-expression signals (FDR < 0.05). We also observed instances where GWAS signals appear to be driven by genetically regulated expression: at 4q24, conditioning on the predicted expression of LRRC37A15P markedly attenuated the TWAS association for Keratin 8 Pseudogene 46 (Fig. 3D), suggesting that LRRC37A15P is the primary effector gene at this locus.

### 3.3. Fine-mapping of TWAS signals using FOCUS

**Figure 4.**
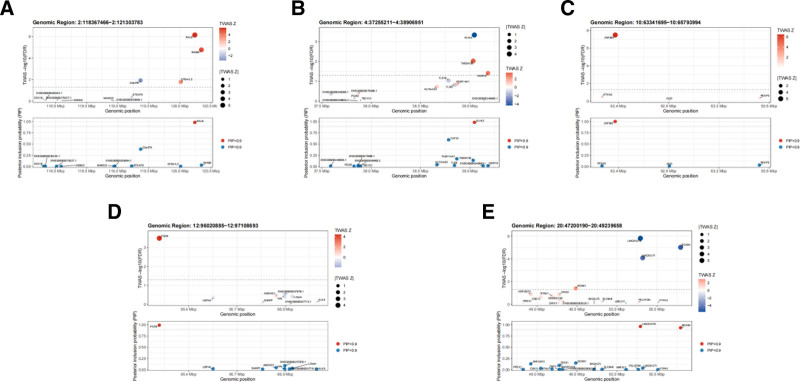
FOCUS fine-mapping results for 5 genomic loci in whole blood. (A–E) FOCUS plots for Chr2:118367466–121303783 (A), Chr4:37255211–38906951 (B), Chr10:63341695–65793994 (C), Chr12:96020885–97108693 (D), and Chr20:47200190–49239658 (E). Each locus shows TWAS significance (−log10 FDR), TWAS *Z*-scores, and posterior inclusion probabilities (PIP) for genes within the locus; genes with PIP > 0.9 are highlighted. FDR = false discovery rate, FUSION = functional summary-based imputation, PIP = posterior inclusion probability.

To prioritize candidate causal genes associated with breast hypertrophy, we performed statistical fine-mapping of TWAS signals using the FOCUS algorithm. This method estimates the posterior inclusion probability for each gene, representing the probability that the gene is causally related to the trait given the observed association. In the analysis of European-ancestry individuals, based on the criteria of FDR < 0.05 and PIP > 0.9, we identified a total of 7 putative causal genes in whole blood tissue (Table 3). The TWAS summary statistics and corresponding PIP values are illustrated in Figure 4. Among these, RALB demonstrated the highest causal probability, with a PIP of 0.98 in GTEx whole blood, suggesting a strong likelihood of causal involvement in breast hypertrophy pathogenesis.

**Table 3:** Statistical fine mapping results: potential causal features.

Location	Gene	SNP weight set	FOCUS PIP
Chr20: 50292719–50292720	LINC01270	GTEx whole blood	0.962
Chr20: 50794893–50794894	BCAS4	GTEx whole blood	0.933
Chr4: 39045038–39045039	KLHL5	GTEx whole blood	0.981
Chr12: 95217481–95217482	FGD6	GTEx whole blood	0.991
Chr2: 120240063–120240064	RALB	GTEx whole blood	0.98
Chr10: 62374191–62374192	ZNF365	GTEx whole blood	1

FOCUS = fine-mapping of causal gene sets, PIP = posterior inclusion probability, SNP = single nucleotide polymorphism.

### 3.4. MAGMA analysis

**Figure 5.**
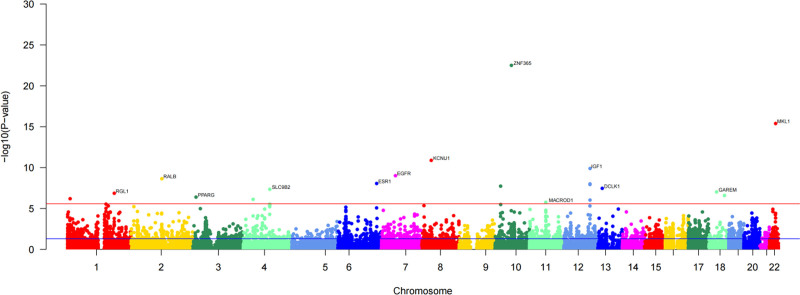
Manhattan plot of MAGMA analysis results. MAGMA = Multi-marker Analysis of GenoMic Annotation.

We performed gene-level association analysis using MAGMA to identify functional genes associated with breast hypertrophy based on GWAS summary statistics. By aggregating the effects of multiple SNPs and accounting for LD, MAGMA improves the power to detect genes with small effect sizes. A total of 189 genes were identified as significantly associated with breast hypertrophy (FDR < 0.05, Table 4), as shown in Figure 5. These genes provide potential targets for further investigation into the genetic architecture and underlying biological mechanisms of breast hypertrophy.

**Table 4:** Breast hypertrophy risk genes identified through gene-based association studies.

GENE	CHR	N	ZSTAT	*P*	FDR
ZNF365	10	265,780	9.86	3.16e−23	6.01e−19
MKL1	22	265,780	8.05	4.09e−16	3.89e−12
KCNU1	8	265,780	6.67	1.30e−11	8.28e−08
IGF1	12	265,780	6.32	1.28e−10	6.09e−07
EGFR	7	265,780	6	9.73e−10	3.71e−06
RALB	2	265,780	5.86	2.31e−09	7.33e−06
ESR1	6	265,780	5.63	8.82e−09	2.35e−05
NUP37	12	265,780	5.61	9.89e−09	2.35e−05
CCDC53	12	265,780	5.58	1.17e−08	2.48e−05
MLLT10	10	265,780	5.5	1.87e−08	3.55e−05
DCLK1	13	265,780	5.39	3.44e−08	5.96e−05
SLC9B2	4	265,780	5.35	4.50e−08	7.14e−05
GAREM	18	265,780	5.21	9.54e−08	1.40e−04
RGL1	1	265,780	5.14	1.37e−07	1.87e−04
BCL2	18	265,780	5.02	2.57e−07	3.26e−04
PPARG	3	265,780	4.93	4.06e−07	4.83e−04
PEX14	1	265,780	4.84	6.37e−07	7.13e−04
KLF3	4	265,780	4.81	7.60e−07	8.04e−04
PARPBP	12	265,780	4.77	9.31e−07	9.33e−04
MACROD1	11	265,780	4.64	1.74e−06	1.65e−03
SEMA6C	1	265,780	4.53	2.91e−06	2.55e−03
SLC9B1	4	265,780	4.53	2.95e−06	2.55e−03
UBE2D3	4	265,780	4.52	3.10e−06	2.57e−03
DNAJC1	10	265,780	4.51	3.27e−06	2.60e−03
CENPE	4	265,780	4.46	4.19e−06	3.19e−03
SGK223	8	265,780	4.44	4.47e−06	3.27e−03
CASQ1	1	265,780	4.42	4.88e−06	3.33e−03
DRAM1	12	265,780	4.42	4.90e−06	3.33e−03
ROCK2	2	265,780	4.38	5.90e−06	3.87e−03
CISD2	4	265,780	4.37	6.12e−06	3.89e−03
C6orf15	6	265,780	4.35	6.96e−06	4.28e−03
CCDC170	6	265,780	4.3	8.46e−06	5.03e−03
TNFAIP8L2	1	265,780	4.28	9.47e−06	5.46e−03
RBMS3	3	265,780	4.26	1.05e−05	5.85e−03
COPS4	4	265,780	4.23	1.17e−05	6.21e−03
DOCK9	13	265,780	4.23	1.17e−05	6.21e−03
THOC5	22	265,780	4.22	1.23e−05	6.34e−03
LSP1	11	265,780	4.21	1.30e−05	6.52e−03
CCHCR1	6	265,780	4.16	1.60e−05	7.81e−03
FLRT1	11	265,780	4.15	1.65e−05	7.82e−03
COL28A1	7	265,780	4.15	1.68e−05	7.82e−03
ARID5B	10	265,780	4.12	1.86e−05	8.45e−03
GABPB2	1	265,780	4.07	2.31e−05	9.86e−03
EFR3B	2	265,780	4.07	2.32e−05	9.86e−03
PSORS1C1	6	265,780	4.07	2.34e−05	9.86e−03
NIPSNAP1	22	265,780	4.07	2.38e−05	9.86e−03
BAZ1A	14	265,780	4.04	2.62e−05	1.01e−02
MSI2	17	265,780	4.04	2.65e−05	1.01e−02
MPC2	1	265,780	4.04	2.68e−05	1.01e−02
TP73	1	265,780	4.04	2.71e−05	1.01e−02
MYOZ1	10	265,780	4.04	2.72e−05	1.01e−02
PLCB3	11	265,780	4.03	2.83e−05	1.03e−02
CDK18	1	265,780	4.02	2.87e−05	1.03e−02
DCAF6	1	265,780	4.02	2.96e−05	1.04e−02
WAC	10	265,780	4	3.12e−05	1.08e−02
CALCRL	2	265,780	3.99	3.28e−05	1.11e−02
PEX19	1	265,780	3.99	3.34e−05	1.12e−02
SORBS1	10	265,780	3.98	3.45e−05	1.13e−02
SSPN	12	265,780	3.97	3.64e−05	1.15e−02
EFCAB8	20	265,780	3.97	3.64e−05	1.15e−02
PCDP1	2	265,780	3.96	3.75e−05	1.17e−02
SGSM3	22	265,780	3.95	3.94e−05	1.19e−02
SKIDA1	10	265,780	3.95	3.95e−05	1.19e−02
TMEM182	2	265,780	3.94	4.08e−05	1.21e−02
TMEM9	1	265,780	3.93	4.21e−05	1.23e−02
SCTR	2	265,780	3.91	4.67e−05	1.35e−02
CPA1	7	265,780	3.9	4.80e−05	1.36e−02
MANBA	4	265,780	3.88	5.22e−05	1.44e−02
POLR3GL	1	265,780	3.88	5.24e−05	1.44e−02
KLHL17	1	265,780	3.87	5.44e−05	1.48e−02
NR2C1	12	265,780	3.85	5.82e−05	1.55e−02
MGAT3	22	265,780	3.85	5.86e−05	1.55e−02
ANTXR1	2	265,780	3.85	5.93e−05	1.55e−02
BRAF	7	265,780	3.85	6.02e−05	1.55e−02
COPA	1	265,780	3.83	6.34e−05	1.61e−02
RP11-552I14.1	12	265,780	3.82	6.57e−05	1.64e−02
FBXO32	8	265,780	3.79	7.50e−05	1.82e−02
ZNF23	16	265,780	3.79	7.55e−05	1.82e−02
ANKRD17	4	265,780	3.79	7.57e−05	1.82e−02
HOPX	4	265,780	3.78	7.76e−05	1.85e−02
STIP1	11	265,780	3.77	8.18e−05	1.87e−02
AC021860.1	4	265,780	3.77	8.19e−05	1.87e−02
WARS2	1	265,780	3.77	8.23e−05	1.87e−02
SMG6	17	265,780	3.77	8.24e−05	1.87e−02
CPA4	7	265,780	3.76	8.50e−05	1.90e−02
BNIPL	1	265,780	3.76	8.57e−05	1.90e−02
SCEL	13	265,780	3.75	8.87e−05	1.94e−02
ATF7IP	12	265,780	3.74	9.29e−05	1.96e−02
CASC10	10	265,780	3.73	9.41e−05	1.96e−02
MYEOV	11	265,780	3.73	9.44e−05	1.96e−02
CLIC1	6	265,780	3.73	9.45e−05	1.96e−02
PSORS1C2	6	265,780	3.73	9.56e−05	1.96e−02
NBPF12	1	265,780	3.73	9.64e−05	1.96e−02
TEX40	11	265,780	3.73	9.69e−05	1.96e−02
ZSWIM4	19	265,780	3.72	9.86e−05	1.98e−02
DRD2	11	265,780	3.71	1.02e−04	2.03e−02
DCAF8	1	265,780	3.7	1.07e−04	2.08e−02
NOC2L	1	265,780	3.7	1.07e−04	2.08e−02
PHIP	6	265,780	3.7	1.08e−04	2.08e−02
ERI1	8	265,780	3.69	1.12e−04	2.14e−02
FERMT3	11	265,780	3.68	1.16e−04	2.19e−02
DCAF8	1	265,780	3.68	1.17e−04	2.19e−02
DOCK10	2	265,780	3.67	1.23e−04	2.27e−02
STEAP4	7	265,780	3.66	1.29e−04	2.35e−02
BAHD1	15	265,780	3.64	1.34e−04	2.42e−02
CDHR4	3	265,780	3.64	1.35e−04	2.42e−02
ANKRD11	16	265,780	3.64	1.36e−04	2.42e−02
LIN54	4	265,780	3.63	1.40e−04	2.47e−02
TRPC4AP	20	265,780	3.62	1.45e−04	2.51e−02
ZFP64	20	265,780	3.62	1.46e−04	2.51e−02
ADSL	22	265,780	3.62	1.46e−04	2.51e−02
NCSTN	1	265,780	3.62	1.49e−04	2.54e−02
CORT	1	265,780	3.61	1.52e−04	2.56e−02
TNFRSF19	13	265,780	3.61	1.54e−04	2.56e−02
FIP1L1	4	265,780	3.61	1.55e−04	2.56e−02
POU5F1	6	265,780	3.6	1.57e−04	2.58e−02
ZNF19	16	265,780	3.6	1.62e−04	2.64e−02
RGS17	6	265,780	3.59	1.66e−04	2.67e−02
SMG7	1	265,780	3.59	1.68e−04	2.68e−02
ADCY9	16	265,780	3.58	1.70e−04	2.69e−02
MSH5-SAPCD1	6	265,780	3.58	1.71e−04	2.69e−02
CHPT1	12	265,780	3.57	1.78e−04	2.77e−02
MSH5	6	265,780	3.57	1.79e−04	2.77e−02
GNPTAB	12	265,780	3.56	1.86e−04	2.85e−02
THAP9	4	265,780	3.56	1.87e−04	2.85e−02
GATSL2	7	265,780	3.55	1.90e−04	2.87e−02
ZEB1	10	265,780	3.55	1.94e−04	2.90e−02
OTUD3	1	265,780	3.55	1.96e−04	2.92e−02
TBC1D16	17	265,780	3.54	1.98e−04	2.92e−02
PPP1R16B	20	265,780	3.54	2.04e−04	2.98e−02
HLA-A	6	265,780	3.53	2.11e−04	3.03e−02
SLC2A9	4	265,780	3.52	2.12e−04	3.03e−02
ARNTL	11	265,780	3.52	2.13e−04	3.03e−02
EDEM2	20	265,780	3.52	2.14e−04	3.03e−02
PEX11B	1	265,780	3.52	2.15e−04	3.03e−02
SPG7	16	265,780	3.51	2.23e−04	3.12e−02
HOOK1	1	265,780	3.5	2.35e−04	3.25e−02
AC010547.9	16	265,780	3.5	2.37e−04	3.25e−02
WFDC13	20	265,780	3.49	2.37e−04	3.25e−02
SLC25A33	1	265,780	3.49	2.39e−04	3.25e−02
LIX1L	1	265,780	3.49	2.43e−04	3.28e−02
SGK494	17	265,780	3.49	2.45e−04	3.28e−02
NDUFAF6	8	265,780	3.48	2.48e−04	3.30e−02
DNMT3B	20	265,780	3.48	2.50e−04	3.30e−02
IREB2	15	265,780	3.47	2.56e−04	3.36e−02
NR2E1	6	265,780	3.47	2.57e−04	3.36e−02
NUDT22	11	265,780	3.46	2.67e−04	3.45e−02
SH2D3C	9	265,780	3.46	2.68e−04	3.45e−02
PTRHD1	2	265,780	3.45	2.84e−04	3.63e−02
HLA-C	6	265,780	3.44	2.92e−04	3.71e−02
TMEM201	1	265,780	3.43	2.97e−04	3.74e−02
FANCA	16	265,780	3.42	3.10e−04	3.87e−02
NAV1	1	265,780	3.42	3.11e−04	3.87e−02
ITIH4	3	265,780	3.41	3.22e−04	3.95e−02
IFT20	17	265,780	3.41	3.24e−04	3.95e−02
SLC25A39	17	265,780	3.41	3.25e−04	3.95e−02
MAP2K6	17	265,780	3.41	3.26e−04	3.95e−02
TFPI	2	265,780	3.4	3.33e−04	3.99e−02
NOL10	2	265,780	3.4	3.35e−04	3.99e−02
IRAK1BP1	6	265,780	3.4	3.37e−04	3.99e−02
RIN3	14	265,780	3.4	3.37e−04	3.99e−02
TSHZ2	20	265,780	3.39	3.54e−04	4.16e−02
PARP10	8	265,780	3.38	3.59e−04	4.20e−02
PLEKHM3	2	265,780	3.38	3.64e−04	4.23e−02
TRMT112	11	265,780	3.38	3.66e−04	4.23e−02
ILF2	1	265,780	3.36	3.85e−04	4.42e−02
TNNT3	11	265,780	3.36	3.91e−04	4.45e−02
FAM193B	5	265,780	3.36	3.95e−04	4.45e−02
MARCKS	6	265,780	3.36	3.95e−04	4.45e−02
TNNC2	20	265,780	3.35	3.98e−04	4.46e−02
CENPO	2	265,780	3.35	4.03e−04	4.48e−02
RNF123	3	265,780	3.35	4.07e−04	4.50e−02
NCF2	1	265,780	3.35	4.10e−04	4.51e−02
PTPN1	20	265,780	3.34	4.17e−04	4.56e−02
COBLL1	2	265,780	3.34	4.24e−04	4.61e−02
CRTC2	1	265,780	3.33	4.29e−04	4.64e−02
CACNA1I	22	265,780	3.33	4.32e−04	4.65e−02
ANKK1	11	265,780	3.33	4.40e−04	4.68e−02
INTS8	8	265,780	3.32	4.43e−04	4.68e−02
TCF19	6	265,780	3.32	4.43e−04	4.68e−02
AC004899.1	7	265,780	3.32	4.57e−04	4.80e−02
AP1G1	16	265,780	3.31	4.58e−04	4.80e−02
HIVEP3	1	265,780	3.31	4.68e−04	4.85e−02
C6orf58	6	265,780	3.31	4.69e−04	4.85e−02
RREB1	6	265,780	3.31	4.73e−04	4.85e−02
FAM46C	1	265,780	3.3	4.76e−04	4.85e−02
CTB-129P6.11	19	265,780	3.3	4.76e−04	4.85e−02
RP5-966M1.6	3	265,780	3.3	4.79e−04	4.85e−02
RNF165	18	265,780	3.3	4.90e−04	4.94e−02

CALCRL = calcitonin receptor-like receptor, FDR = false discovery rate, KRT8P46 = keratin 8 pseudogene 46, LRRC37A15P = leucine rich repeat containing 37 member A15 pseudogene.

### 3.5. Mendelian randomization and colocalization analysis

**Figure 6.**
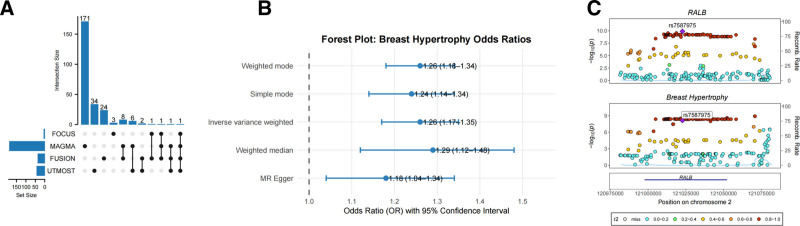
Mendelian randomization and colocalization analyses of a key susceptibility gene for breast hypertrophy. (A) UpSet plot showing the overlap of candidate genes identified by UTMOST, FUSION, MAGMA, and FOCUS analyses. (B) Forest plot of Mendelian randomization results assessing the causal relationship between RALB expression and breast hypertrophy. (C) Colocalization analysis of the RALB locus. The x-axis represents genomic position (ordered by chromosome coordinates), and the y-axis shows statistical significance as −log10(*P*) values. Higher values indicate stronger associations. Stacked bar plots illustrate the relative contributions of different variants at this locus, with bar height and color denoting the magnitude and relevance of each signal. FOCUS = fine-mapping of causal gene sets, FUSION = functional summary-based imputation, MAGMA = Multi-marker Analysis of GenoMic Annotation, RALB = RAS like proto-oncogene B, UTMOST = unified test for molecular signatures.

To enhance the robustness and consistency of our findings, we performed an integrative analysis by combining cross-tissue results from UTMOST with candidate genes identified by FUSION and MAGMA. This multi-method cross-validation approach led to the identification of a key candidate gene, RALB (Fig. 6A). Notably, RALB demonstrated statistically significant associations across multiple analytical platforms, suggesting a consistent genetic signal and potential biological relevance across different tissues and physiological conditions.

A two-sample Mendelian randomization (MR) analysis was conducted to investigate the potential causal effect of RALB expression on breast hypertrophy. This analysis integrated eQTL data for RALB from whole blood with GWAS summary statistics for breast hypertrophy. All IVs (SNPs) used in the analysis were strong (*F*-statistic > 10), supporting their validity. The MR results revealed a significant positive causal association between increased RALB expression and the risk of breast hypertrophy (*P* < .001; odds ratio = 1.26; 95% confidence interval: 1.17–1.35) (Fig. 6B). These findings suggest a potential role of RALB in the etiology of breast hypertrophy and warrant further investigation into its underlying biological mechanisms.

To evaluate the potential sharing of GWAS and eQTL signals, we performed colocalization analysis with a 50 kb window. In whole blood, the susceptibility gene RALB, located at 2q14.3, showed strong evidence of colocalization with signals associated with breast hypertrophy. The posterior probability for hypothesis 4 (PP4) was 0.922 (Fig. 6C), exceeding the commonly accepted threshold of 0.8, indicating a high likelihood that the GWAS and eQTL signals originate from a shared causal variant. This finding supports a potential causal role of RALB in the development of breast hypertrophy.

## 4. Discussion

In this study, we performed a comprehensive cross-tissue TWAS integrating GTEx eQTL data with FinnGen GWAS summary statistics, identifying RALB as a robust candidate gene causally associated with breast hypertrophy. The association was consistently supported across multiple methodologies, including single-tissue TWAS, fine-mapping, gene-level analysis, Mendelian randomization, and colocalization, underscoring the reliability of our findings.

RALB, a member of the Ras superfamily of small GTPases, is implicated in key cellular processes such as inflammatory signaling, vesicle trafficking, and cell proliferation.^[23,24]^ Mechanistically, RALB modulates inflammatory cytokine expression through interaction with RalA Binding Protein 1 and activation of the TANK-Binding Kinase 1 (TBK1)–Interferon Regulatory Factor 3 pathway, potentially contributing to chronic low-grade inflammation and tissue remodeling in breast hypertrophy.^[25]^ Additionally, its role in vesicle trafficking via the exocyst complex influences secretion of growth factors like epidermal growth factor and vascular endothelial growth factor, which may enhance local proliferation and angiogenesis.^[26]^ Moreover, RALB’s involvement in the Phosphoinositide 3-Kinase–protein kinase B-mechanistic target of rapamycin (mTOR) signaling pathway further supports its contribution to glandular and adipose tissue expansion characteristic of the disease.^[27,28]^ Although not directly hormone-responsive, RALB might affect hormonal sensitivity indirectly through modulation of the extracellular milieu or vascular perfusion. Emerging evidence also suggests that RALB is involved in breast cancer progression through similar mechanisms, including cell migration and immune evasion,^[29]^ highlighting its broader role in mammary tissue biology.

Beyond RALB, we also identified additional independent TWAS signals at loci such as CALCRL and LRRC37A15P. CALCRL encodes a receptor component for adrenomedullin and calcitonin gene-related peptide, hormones implicated in angiogenesis and vasodilation.^[30–32]^ CALCRL may influence tissue perfusion and promote hypertrophy of stromal and adipose components. LRRC37A15P, although classified as a pseudogene, may exert regulatory functions via mechanisms such as competing endogenous RNA networks or chromatin remodeling,^[33]^ though further studies are needed to elucidate its role.

The integration of multiple TWAS methodologies enhanced the robustness of our findings. UTMOST leverages cross-tissue information to improve statistical power, while FUSION provides tissue-specific validation, particularly in whole blood, which serves as a high-quality eQTL reference. The convergence of evidence across both cross-tissue and single-tissue approaches underscores the biological relevance of the identified genes.

Furthermore, our MR and colocalization analyses provided strong support for a causal relationship between genetically predicted gene expression and breast hypertrophy risk. We applied stringent criteria for selecting IVs and conducted sensitivity analyses (e.g., MR-Egger, Mendelian Randomization Pleiotropy RESidual Sum and Outlier) to account for horizontal pleiotropy and potential confounding, thereby increasing confidence in our causal inference.

Drawing on convergent lines of evidence, we nominate RALB as a plausible effector gene for breast hypertrophy. In the near term, RALB expression levels or genetically inferred RALB activity could be explored as biomarkers to refine risk stratification. From an intervention standpoint, given that RALB participates in vesicle trafficking via the exocyst and signals through the TBK1–Interferon Regulatory Factor 3 and Phosphoinositide 3-Kinase–protein kinase B-mTOR pathways, more feasible pharmacologic entry points at this stage are likely downstream actionable nodes rather than direct inhibition of RALB itself. Testable preclinical hypotheses include small-molecule strategies that disrupt RALB–effector interfaces (e.g., RALB–RalA Binding Protein 1) or modulate TBK1/mTOR signaling. Importantly, because our primary inferences relied on whole-blood eQTLs, breast-tissue validation (e.g., expression profiling and perturbation experiments such as overexpression/knockdown, CRISPR-mediated editing, and organoid or single-cell systems) will be essential prior to clinical translation.

Despite these strengths, several limitations warrant consideration. First, our main analyses did not incorporate breast-specific eQTL reference panels; instead, we relied on cross-tissue and/or whole-blood eQTL resources for TWAS. This may underestimate truly tissue-specific effects in the breast and limits direct inference on gene regulation within mammary tissue. Second, by design, TWAS cannot fully distinguish a causal gene from nearby co-regulated genes at the statistical level; functional studies – such as CRISPR perturbation, single-cell and spatial transcriptomics, allele-specific expression, and reporter assays – are required to establish causality. Finally, our findings are based on individuals of European ancestry, and their generalizability to other populations requires evaluation in multi-ethnic, independent cohorts.

In conclusion, through an integrative TWAS framework, we identified RALB as a promising causal gene in the genetic architecture of breast hypertrophy. These results offer new insights into the molecular mechanisms underlying breast hypertrophy and suggest potential targets for future therapeutic or preventive strategies. Further functional validation and replication in diverse populations will be essential to clarify the role of RALB and other candidate genes in mammary tissue development and hypertrophy.

## Acknowledgments

We thank the participants and investigators of the FinnGen and GTEx projects for making their data publicly available.

## Author contributions

**Conceptualization:** Jingshuang Chen, Zujian Hu, Hua Luo.

**Data curation:** Junwei Gu, Jingshuang Chen, Zujian Hu, Hua Luo.

**Formal analysis:** Junwei Gu, Jingshuang Chen, Zujian Hu.

**Funding acquisition:** Zujian Hu.

**Investigation:** Junwei Gu, Jingshuang Chen.

**Methodology:** Junwei Gu, Jingshuang Chen.

**Project administration:** Junwei Gu, Jingshuang Chen.

**Resources:** Junwei Gu, Jingshuang Chen.

**Software:** Junwei Gu, Jingshuang Chen.

**Supervision:** Zujian Hu, Hua Luo.

**Validation:** Hua Luo.

**Visualization:** Hua Luo.

**Writing – original draft:** Junwei Gu, Jingshuang Chen.

**Writing – review & editing:** Zujian Hu, Hua Luo.

## Supplementary Material


