# Supplementary material for: Cross-tissue transcriptome-wide association and Mendelian randomization identify RALB as a susceptibility gene for breast hypertrophy
Source: Medicine (Baltimore). 2025 Nov 14;104(46):e45872. doi: 10.1097/MD.0000000000045872 (PMC12622666; doi:10.1097/MD.0000000000045872)
Supplement: Supplementary file 1 [file medi-104-e45872-s001.docx]

**Supplementary Table 1. Significant genes associated with breast hypertrophy risk identified by cross-tissue TWAS (p < 0.05)**

| gene | chr | test_score | p_value |
| --- | --- | --- | --- |
| HS3ST1 | 4 | 4.73124271023226 | 0.0157124766537837 |
| UPP2 | 2 | 3.76770880809404 | 0.0200141751430805 |
| NCAPH2 | 22 | 4.27259958287918 | 0.0376773617813924 |
| IKZF2 | 2 | 4.72400297435353 | 0.01076262261664 |
| TSSC1 | 2 | 3.56586166544801 | 0.0407918927200203 |
| AGA | 4 | 2.59001691690139 | 0.0173005839760935 |
| CALCRL | 2 | 9.78050987908026 | 4.175321363864e-05 |
| ZC3H15 | 2 | 9.42263097183057 | 8.83696262524714e-05 |
| MGAT4A | 2 | 3.96594706388916 | 0.0229046041690461 |
| WDR1 | 4 | 6.72413805972495 | 0.000840835536201512 |
| GLI2 | 2 | 5.1998384757372 | 0.00425474072511356 |
| TNS1 | 2 | 10.7662754105641 | 2.0170895527416e-05 |
| SCTR | 2 | 3.46533582726221 | 0.0198318505640051 |
| CXCL2 | 4 | 4.74580485431444 | 0.0105247735363251 |
| AFP | 4 | 3.776918917 | 0.0290110424931255 |
| COBLL1 | 2 | 9.08310184735586 | 7.6281976507997e-05 |
| APOB | 2 | 3.43205313699891 | 0.0401426313795782 |
| NCOA1 | 2 | 5.87216313792078 | 0.000724108566527937 |
| EFR3B | 2 | 5.23874953581169 | 0.00955764401234593 |
| KIF3C | 2 | 6.05431340835453 | 0.00286095500404682 |
| RAB10 | 2 | 4.78703042642576 | 0.00853519674684278 |
| GCKR | 2 | 3.92196346945182 | 0.0193697022061107 |
| MAPRE3 | 2 | 2.63111414606276 | 0.0484712273912566 |
| PTPN4 | 2 | 7.48322249951066 | 0.000391478255082678 |
| TRMT2A | 22 | 3.86348958080077 | 0.0220622885650362 |
| CRYBB2P1 | 22 | 7.85732330370413 | 0.000170231282605515 |
| GRK3 | 22 | 7.54857382836575 | 0.00021461383951682 |
| PATZ1 | 22 | 3.40128059976115 | 0.0232781477254069 |
| HSCB | 22 | 3.79817041962912 | 0.0101508673126792 |
| RHBDD3 | 22 | 5.27256719768208 | 0.00180399113204777 |
| THOC5 | 22 | 4.77502795802457 | 0.00294266296612589 |
| RASD2 | 22 | 4.53271955567507 | 0.0158412976417057 |
| PDGFB | 22 | 4.02302700475581 | 0.0211402270427324 |
| ASCC2 | 22 | 4.64158964568869 | 0.00517794364815882 |
| MIEF1 | 22 | 4.4855616715976 | 0.0235332297584259 |
| GRAP2 | 22 | 6.05256906479972 | 0.00257701177801217 |
| TNRC6B | 22 | 4.3468018257674 | 0.00970263030480534 |
| SGSM3 | 22 | 7.60013958880437 | 0.000733482717210632 |
| CSF2RB | 22 | 4.69009170575217 | 0.0258345519413892 |
| SLC25A17 | 22 | 4.74279332977094 | 0.00573843076043146 |
| L3MBTL2 | 22 | 2.59625165116866 | 0.0401243167767575 |
| NOL4 | 18 | 6.11006388967125 | 0.00285821627390603 |
| TMEM33 | 4 | 4.31417304026844 | 0.0144620845827304 |
| SLAIN2 | 4 | 3.6920273034206 | 0.0246550040536722 |
| CWH43 | 4 | 4.76254090607789 | 0.0150832316082127 |
| USP46 | 4 | 4.98251663918811 | 0.00833696573710163 |
| SMR3A | 4 | 2.4323462564133 | 0.0385651992129299 |
| NFKB1 | 4 | 7.34156543141516 | 0.000724345297091977 |
| MANBA | 4 | 7.46169909657194 | 0.000690848672266431 |
| UBE2D3 | 4 | 11.5052171771471 | 3.53656005269709e-06 |
| SLC2A9 | 4 | 6.24758690444243 | 0.00111690408294085 |
| CLNK | 4 | 4.78267947684618 | 0.0133680466366212 |
| ZAP70 | 2 | 2.80634678872136 | 0.0213663793950544 |
| POMC | 2 | 3.93648057535393 | 0.00709641123008709 |
| MPV17 | 2 | 4.83275493273362 | 0.0171543707126164 |
| NRBP1 | 2 | 2.4592943509528 | 0.032229431188381 |
| ITGB6 | 2 | 2.89889095461596 | 0.0357604889507642 |
| ITGA4 | 2 | 3.79094336347031 | 0.0434175973578239 |
| SNX17 | 2 | 3.93212481097549 | 0.0380320946855975 |
| PPM1G | 2 | 2.85863204981996 | 0.0270514528151525 |
| MOGS | 2 | 2.7843177061724 | 0.0176418943162556 |
| GRB14 | 2 | 3.40436591969009 | 0.0247517374833419 |
| CCDC88A | 2 | 6.10655640871451 | 0.0019921327282264 |
| EFEMP1 | 2 | 7.38812886197847 | 0.000464447886182162 |
| IGFBP5 | 2 | 4.80041692017812 | 0.00985669558988256 |
| PROC | 2 | 5.69585546247542 | 0.00681673702897234 |
| ODC1 | 2 | 4.6843211625093 | 0.0108555390693531 |
| KYNU | 2 | 6.0912986565561 | 0.00378278396590692 |
| ORC4 | 2 | 5.36363854932166 | 0.00430678320050859 |
| MREG | 2 | 3.12175045036912 | 0.0363263472762163 |
| TNP1 | 2 | 9.99646557719787 | 4.09901132205626e-05 |
| STBD1 | 4 | 3.79660354134505 | 0.018562058375278 |
| LIAS | 4 | 5.14718508283378 | 0.00347971052903862 |
| ACVR2A | 2 | 6.06292788863725 | 0.00104088328340113 |
| AAMP | 2 | 3.50540095290721 | 0.0217413763564892 |
| KDR | 4 | 3.63653551207046 | 0.0181648551497206 |
| ENAM | 4 | 3.33943801327517 | 0.0436476960447504 |
| SEC14L4 | 22 | 2.99888351021666 | 0.0259247078401955 |
| ROCK2 | 2 | 4.24823066306482 | 0.0253733674051777 |
| TGFBRAP1 | 2 | 3.77232039539292 | 0.0220019283022702 |
| GCC2 | 2 | 2.61029179333735 | 0.0386186593711075 |
| 45723 | 2 | 4.38868996705157 | 0.0194608772436513 |
| TANK | 2 | 3.85142239529198 | 0.0407923887040432 |
| FHDC1 | 4 | 3.6063087392785 | 0.0270142359535613 |
| SELENOI | 2 | 9.15713256989424 | 9.30372993892536e-05 |
| ADCY3 | 2 | 67.2679568083181 | 0.0170357282772923 |
| PNPT1 | 2 | 4.94253221018658 | 0.020923734015635 |
| SIX3 | 2 | 4.31112242156595 | 0.00924051492357925 |
| CENPO | 2 | 6.54857154071539 | 0.000958977239573278 |
| TRIM54 | 2 | 37.3079660371602 | 0.0260222326044858 |
| DTNB | 2 | 4.71118019409359 | 0.00803951873227893 |
| SCARB2 | 4 | 4.39347517067634 | 0.0129766427466047 |
| CENPE | 4 | 13.5718959735451 | 4.0390627509268e-07 |
| SLC39A8 | 4 | 8.01918113883346 | 0.00022089607499709 |
| MTTP | 4 | 4.2073983873247 | 0.0330577119506129 |
| RNF185 | 22 | 3.09828792707779 | 0.0248253227802859 |
| ATP6V1C2 | 2 | 7.35321772541338 | 0.000813470507767367 |
| ASXL2 | 2 | 7.00401394531001 | 0.000838224366348617 |
| MEIS1 | 2 | 3.24680655992468 | 0.0445057869914207 |
| RALB | 2 | 9.80411938324008 | 3.04585317610906e-05 |
| TMEM177 | 2 | 8.6167610340574 | 0.000133622937057876 |
| LNPK | 2 | 2.97035945572566 | 0.0194066199969355 |
| GULP1 | 2 | 4.82822731396983 | 0.00805636004226063 |
| NYAP2 | 2 | 7.99919051797993 | 0.00029975025393425 |
| EPHA5 | 4 | 3.56081229312329 | 0.0329539373906321 |
| SLC10A4 | 4 | 4.00861450729477 | 0.00961643055768691 |
| SCD5 | 4 | 3.32189493365799 | 0.0275308879018964 |
| ENOPH1 | 4 | 7.10625034787048 | 0.000749156037596843 |
| CISD2 | 4 | 10.4293403191608 | 6.40586810929378e-06 |
| ANO7 | 2 | 2.41086175910526 | 0.0358659142225283 |
| VEGFC | 4 | 4.62020131898257 | 0.0123307275654982 |
| TKTL2 | 4 | 4.84320408310073 | 0.0112064585896458 |
| PIGF | 2 | 3.41580064752434 | 0.0243308768626097 |
| MZT2B | 2 | 4.80123138705742 | 0.0104357360856554 |
| RIT2 | 18 | 5.21000721316324 | 0.00495851931208602 |
| HAUS1 | 18 | 3.68347334673345 | 0.00746615893608582 |
| STK32B | 4 | 3.16411489263925 | 0.0472757026273961 |
| CXXC1 | 18 | 4.56321671344872 | 0.0113732083298607 |
| DBI | 2 | 11.4390867341437 | 9.04805488322058e-06 |
| ADAMTS3 | 4 | 3.90916560221723 | 0.0238371580827905 |
| GAREM2 | 2 | 4.48248350322609 | 0.006287024460358 |
| CIB4 | 2 | 4.12240205064826 | 0.0215622290732092 |
| KRTCAP3 | 2 | 4.27348068458986 | 0.00631265512736601 |
| PEX13 | 2 | 3.6805140262335 | 0.0248887329459431 |
| LRRTM1 | 2 | 3.6979092500764 | 0.0274488966034533 |
| CFAP36 | 2 | 4.3629009923596 | 0.0198418720362721 |
| NUP35 | 2 | 3.03418603438399 | 0.046339002607157 |
| CCDC138 | 2 | 3.80122100812573 | 0.0469274414428651 |
| WDCP | 2 | 5.13933638518932 | 0.00764448000284212 |
| VSNL1 | 2 | 3.13069295670015 | 0.045525676035152 |
| CFAP221 | 2 | 6.45633313868004 | 0.000656178902564752 |
| INHBB | 2 | 3.61744454650216 | 0.020023134922023 |
| SMARCAD1 | 4 | 45.5282095837644 | 0.0269152785029781 |
| MSX1 | 4 | 3.71764172279804 | 0.0162933611636052 |
| C1QTNF7 | 4 | 4.11839526055401 | 0.0149208590427143 |
| BMP10 | 2 | 3.24755208153239 | 0.0299550089042061 |
| ARHGAP25 | 2 | 7.55200775077822 | 0.000298573104738797 |
| DCAF16 | 4 | 4.2674668442483 | 0.00801035899451586 |
| GABRG1 | 4 | 3.66649297820045 | 0.0207068744277259 |
| MRPS18C | 4 | 23.9448426895038 | 1.84200432684634e-11 |
| CCKAR | 4 | 4.336011573 | 0.0161332540536802 |
| NKX6-1 | 4 | 18.0029133185143 | 9.21352516503049e-09 |
| CDS1 | 4 | 9.04059106073801 | 0.000141656914041444 |
| WDFY3 | 4 | 15.1897725970995 | 2.70870180285598e-07 |
| COX18 | 4 | 5.98085837426648 | 0.0039656778935 |
| CXCL3 | 4 | 4.09395115397336 | 0.0254656337548747 |
| CXCL5 | 4 | 4.97578247084311 | 0.00783811608817364 |
| PPBP | 4 | 3.44220346647533 | 0.0300569220558302 |
| RCHY1 | 4 | 3.7068971589452 | 0.0168731954389784 |
| ZNF513 | 2 | 5.34962806525697 | 0.00315931430267757 |
| SPDYA | 2 | 4.30826843026705 | 0.0151110646574526 |
| WDR43 | 2 | 5.59652636432308 | 0.012759731525432 |
| SLC9B1 | 4 | 7.0561165453007 | 0.00256007211341391 |
| SLC9B2 | 4 | 9.41825575757315 | 9.54199384979315e-05 |
| BDH2 | 4 | 8.20718522235362 | 5.30038826810353e-05 |
| NDST3 | 4 | 3.49304740434737 | 0.0366107113749666 |
| ARFIP1 | 4 | 2.37343510657917 | 0.0258902798128482 |
| SERPINB7 | 18 | 3.72205257584114 | 0.032497511733808 |
| ACAA2 | 18 | 4.47097855763479 | 0.0113915435559275 |
| ZCCHC4 | 4 | 3.90810970538548 | 0.0158549549096749 |
| IRF2 | 4 | 5.03485039473672 | 0.00983374506726664 |
| ATG4B | 2 | 2.34071892648966 | 0.0475567618922987 |
| ADAM29 | 4 | 4.27715748605246 | 0.017471999130079 |
| MFF | 2 | 3.7578601446907 | 0.0122989770792664 |
| NTSR2 | 2 | 9.30162388776221 | 7.79766941763072e-05 |
| E2F6 | 2 | 3.26502741956522 | 0.0349957163810593 |
| CXCL10 | 4 | 4.06936676939998 | 0.0152499500394439 |
| SLC38A11 | 2 | 2.73738859441055 | 0.0423742201649453 |
| TACR3 | 4 | 13.1257439696048 | 1.95204553676831e-06 |
| MAP3K2 | 2 | 2.49689300018272 | 0.0456316728067807 |
| TIGD4 | 4 | 3.33608916500572 | 0.0112934094465953 |
| RNF150 | 4 | 4.84134606615017 | 0.00664094611175214 |
| HOXD12 | 2 | 3.82339052882154 | 0.017345714378264 |
| AGXT | 2 | 2.79792021135946 | 0.0463372450914601 |
| HPSE | 4 | 4.79937800295978 | 0.00428902156659128 |
| COMMD1 | 2 | 5.19069970249396 | 0.0288759071646958 |
| ADGRF3 | 2 | 3.37909926410593 | 0.0182290428768678 |
| UBXN2A | 2 | 3.6385046463744 | 0.0150833190065713 |
| TLR10 | 4 | 9.27564083273921 | 0.000306421885430064 |
| TLR6 | 4 | 5.3017026985174 | 0.0033809612703315 |
| THAP6 | 4 | 5.5865262504699 | 0.00533342225232902 |
| BTC | 4 | 2.98343931907885 | 0.0396536285570233 |
| PTPN2 | 18 | 3.02582844464217 | 0.044842807642543 |
| ENTHD1 | 22 | 3.40254325400327 | 0.0448915394954381 |
| CCDC121 | 2 | 4.38696809908559 | 0.0255439028329613 |
| NAP1L5 | 4 | 5.5718182204479 | 0.00135769356929794 |
| C2orf73 | 2 | 4.58542269543779 | 0.00504967939783929 |
| TSPYL6 | 2 | 2.99301071972462 | 0.0344842569761616 |
| H3F3AP6 | 4 | 3.46093247971528 | 0.0473286969124163 |
| AMBN | 4 | 3.82980943786149 | 0.0250984891224839 |
| LINC00305 | 18 | 2.72682015835787 | 0.0443208564919988 |
| FAR2P1 | 2 | 2.87679458864172 | 0.048529380885554 |
| MCFD2 | 2 | 2.5216908823635 | 0.0345037798665323 |
| GSX2 | 4 | 4.602889129 | 0.0124614771174666 |
| RP4-539M6.14 | 22 | 7.11001093938432 | 0.000836822913805713 |
| CRYGB | 2 | 2.66720139904088 | 0.0353666559664917 |
| FIGLA | 2 | 3.74840399702006 | 0.020353893511396 |
| KREMEN1 | 22 | 6.48429222735199 | 0.00109964044200106 |
| NIPSNAP1 | 22 | 5.07866229521108 | 0.00156798256621127 |
| SCFD2 | 4 | 4.39975185602172 | 0.00745592621042268 |
| KCNK12 | 2 | 4.09424968570475 | 0.0191362177171801 |
| PIWIL3 | 22 | 2.30029586222284 | 0.0214314639446584 |
| PTRHD1 | 2 | 10.7329192447036 | 2.45945483089427e-05 |
| NT5C1B | 2 | 5.60632982108458 | 0.00286111556881508 |
| RGPD2 | 2 | 2.68199823018136 | 0.0360483869321678 |
| DRG1 | 22 | 4.6065728584529 | 0.0113775423337211 |
| PRR30 | 2 | -2.898394188 | 0.00375078847202791 |
| AC007731.1 | 22 | 2.42464991974791 | 0.0153231550555942 |
| C22orf34 | 22 | 4.4359736436802 | 0.00352593447358207 |
| PRSS48 | 4 | 2.88008242403129 | 0.048022702549372 |
| ALKAL2 | 2 | 4.09226473567124 | 0.0191806856838892 |
| TOGARAM2 | 2 | 3.29876962377526 | 0.0158251991913992 |
| SPATS2L | 2 | 3.62831420710021 | 0.0352207747779995 |
| GREB1 | 2 | 3.65486310881641 | 0.0192475839572472 |
| ARL9 | 4 | 10.3858456608082 | 5.4411321780079e-05 |
| POTEF | 2 | 6.70718856219119 | 0.00183413964950629 |
| AC004057.1 | 4 | 5.50192604113792 | 0.00616591574194536 |
| MAML3 | 4 | 3.91253747754327 | 0.0250279428468662 |
| C1D | 2 | 4.48416281576888 | 0.00965838461214763 |
| FAM114A1 | 4 | 2.78578295786939 | 0.0313174678791047 |
| C4orf22 | 4 | 3.51913675806562 | 0.0486258564788791 |
| SFI1 | 22 | 3.19798298665339 | 0.012654907058938 |
| SDAD1 | 4 | 3.43943754077647 | 0.0441357789286915 |
| PABPC1P2 | 2 | -2.785239488 | 0.00534882013024614 |
| AC015969.3 | 2 | 3.21402154106099 | 0.0329267535624389 |
| AC013472.3 | 2 | 4.33448878223373 | 0.0130137124395091 |
| IGKJ3 | 2 | 3.01300176987249 | 0.049259584006771 |
| IGKJ2 | 2 | 8.62321379847477 | 0.000127928425757928 |
| IGKV4-1 | 2 | 5.8378140015292 | 0.00286044593110146 |
| IGKV3D-11 | 2 | 4.3766665702504 | 0.0360344657695317 |
| IGKV1D-42 | 2 | 3.50022895840401 | 0.0267409155108525 |
| IGLV3-22 | 22 | 3.73841254543306 | 0.0232395996325109 |
| IGLJ2 | 22 | 3.58887063821672 | 0.0320974786552753 |
| IGLJ3 | 22 | 6.92800861690538 | 0.00171690231100829 |
| IGLC7 | 22 | 3.64412464287193 | 0.0345262146881927 |
| AC010731.6 | 2 | -2.344566393 | 0.0190492186517173 |
| BTF3L4P2 | 2 | 5.12424818696191 | 0.00806203274545037 |
| RP1-29C18.9 | 22 | 6.78624720740073 | 0.00304214929995883 |
| SLC25A14P1 | 4 | 5.1452150885548 | 0.00301191947748281 |
| PPP1CB | 2 | 2.94423591976573 | 0.0142648805452734 |
| RP11-12M9.4 | 22 | 5.4076392884385 | 0.00429795526094945 |
| RPL7P17 | 4 | 4.74654744497528 | 0.0128770211021474 |
| RP11-247I13.3 | 22 | 3.58242515439053 | 0.0254885473724238 |
| SDC4P | 22 | 8.19732872229256 | 0.000115944148235791 |
| GCC2-AS1 | 2 | 1.98817264005046 | 0.0497850243588788 |
| RAD51AP2 | 2 | 2.01664836214368 | 0.0470727584039521 |
| RP11-115L11.1 | 4 | 2.78369528944941 | 0.0215347487587196 |
| DCDC2C | 2 | 3.34636433278934 | 0.0248390432737788 |
| GRXCR1 | 4 | -2.610734151 | 0.00903480980713854 |
| LRRC37A14P | 22 | -2.964177615 | 0.00303493059151845 |
| LINC01793 | 2 | 4.6064989426085 | 0.0373951313921521 |
| AC112721.2 | 2 | 2.20536623897948 | 0.0489288499633386 |
| RN7SKP169 | 22 | -1.99778407 | 0.0457400751366398 |
| MTND5P28 | 2 | 8.42245187484383 | 0.000111038579701073 |
| LINC01120 | 2 | 2.76912548436501 | 0.00562069827288534 |
| AC008073.9 | 2 | 3.89835259580891 | 0.0123928025411528 |
| YES1P1 | 22 | 6.2134086590472 | 0.0010666818658821 |
| AC007319.1 | 2 | 7.80803763167603 | 0.000182445528856268 |
| AC009506.1 | 2 | 3.02792167354653 | 0.0225921243219072 |
| DNAJC27-AS1 | 2 | 3.86939201228319 | 0.00886483071260436 |
| AC019181.3 | 2 | 6.8463534750566 | 0.00105666149880101 |
| KRT18P39 | 2 | 3.99200247819961 | 0.0132074320181548 |
| BCRP1 | 22 | 3.62395783908943 | 0.0125741495133108 |
| AC092484.1 | 2 | 8.7195455610567 | 4.97313575902325e-05 |
| TPI1P4 | 4 | 3.27202225276522 | 0.043590291136419 |
| AC005237.4 | 2 | 4.18328501036317 | 0.00728063829149139 |
| IGKV6D-21 | 2 | 3.25425026839492 | 0.043216846298811 |
| RP3-370M22.8 | 22 | 3.95470122952897 | 0.0281423749334678 |
| AC079163.1 | 2 | 2.89017605781555 | 0.00385026126799823 |
| AC105402.3 | 2 | 11.1154459796109 | 0.0311190910492266 |
| TMEM185B | 2 | 8.0232645819502 | 5.06195463058079e-05 |
| TEX41 | 2 | 3.72831103836989 | 0.0265467017055442 |
| AC097724.3 | 2 | 7.7176972819158 | 0.00061746217287606 |
| ERVMER34-1 | 4 | 6.55100320927734 | 0.00179485945800695 |
| AC007557.3 | 2 | 4.77960012816086 | 0.00357208835571388 |
| RP11-533F5.2 | 4 | -2.318501137 | 0.0204221012566042 |
| AC073636.1 | 2 | 4.92472276775613 | 0.00612331646035857 |
| IL21-AS1 | 4 | 4.18270210814939 | 0.0134965719979427 |
| AC009502.4 | 2 | 1.96297114029513 | 0.0496495275153866 |
| MTND3P9 | 2 | 2.77115076137158 | 0.0055858557368031 |
| MTCO3P43 | 2 | 7.20469880648505 | 0.000321070114209232 |
| AC010967.2 | 2 | 2.76796673374378 | 0.0213118316087765 |
| AC097713.3 | 2 | 3.46086671851056 | 0.0423288949580422 |
| AC016717.1 | 2 | 7.66596617553738 | 0.000394391840005448 |
| AC074019.1 | 2 | 1.97618046606436 | 0.0481343414673979 |
| AC007381.3 | 2 | 5.35418715867451 | 0.00536472071492822 |
| RPL23AP26 | 2 | 2.54190805315455 | 0.0110249171871855 |
| AC096554.1 | 2 | 2.08641479539059 | 0.0369410659266109 |
| AC009495.4 | 2 | 3.7037813739614 | 0.0343698851118482 |
| AC069154.4 | 2 | 2.76458684815911 | 0.0300625606820417 |
| PKP4P1 | 2 | 2.95436574612983 | 0.00313312442056474 |
| CXCR2P1 | 2 | 3.86872306927565 | 0.0255545631137079 |
| LRRC37A15P | 4 | 13.0576784811383 | 4.53058745963908e-07 |
| FAM92A1P2 | 4 | -2.046132783 | 0.0407433083206833 |
| AC013472.4 | 2 | 9.79271657536456 | 8.3407319759532e-05 |
| AC114755.2 | 2 | -3.091075275 | 0.00199433068153589 |
| LINC01381 | 2 | 3.14833902990573 | 0.021954809910159 |
| RP11-681L4.1 | 2 | 15.4042891107921 | 0.0258468954763905 |
| LINC00486 | 2 | 4.01595232253367 | 0.0365328526912602 |
| RPL7L1P9 | 2 | 6.53651413485998 | 0.00148719431332245 |
| LINC01911 | 2 | 2.5551342050523 | 0.0106146863255043 |
| AC007238.1 | 2 | 8.47123034076744 | 0.000178403427588347 |
| AC116609.3 | 2 | -2.06670432 | 0.0387620238947206 |
| KLF2P1 | 2 | 3.76689753031909 | 0.0378096797814032 |
| AC104634.3 | 2 | 3.46111826538828 | 0.0426390012800365 |
| MORF4 | 4 | 3.60548341889626 | 0.0209018843891982 |
| KRT18P63 | 4 | 3.46652524974121 | 0.0279398013847235 |
| RP11-57G22.1 | 18 | 2.11145563317323 | 0.0347331675444839 |
| RN7SL28P | 4 | 2.16942070234257 | 0.0300507586499856 |
| RP11-15B17.1 | 4 | 4.74840240091432 | 0.00602523374084474 |
| CXXC4-AS1 | 4 | 3.34234058683277 | 0.0327923375412031 |
| RP11-10L12.4 | 4 | 9.71483086691362 | 1.55581245521175e-05 |
| RP11-231C18.1 | 4 | 2.91626750636652 | 0.0289871318204055 |
| CCNL2P1 | 4 | 3.85567422650049 | 0.0487410450820268 |
| FOSL1P1 | 4 | 3.84017761291789 | 0.0440227032768403 |
| LINC01085 | 4 | 2.90966920964686 | 0.0238403971409173 |
| RP11-377G16.2 | 4 | 3.26614244992876 | 0.0435486024612231 |
| RP11-412P11.1 | 4 | 2.4003156761443 | 0.0163809383660735 |
| KRT8P46 | 4 | 12.7205308111459 | 7.66949499797498e-07 |
| RP11-358D17.1 | 4 | 3.07829301666249 | 0.0432627726338855 |
| RP11-593F5.2 | 4 | -3.266485367 | 0.00108891435565273 |
| RP11-774O3.2 | 4 | 1.64952915588526 | 0.0368397985891425 |
| RP11-436F23.1 | 4 | 6.3807383628606 | 0.001239438514546 |
| ZEB2P1 | 4 | 2.76781740870895 | 0.0293308063031672 |
| RP11-703G6.1 | 4 | -2.419754431 | 0.0155309914372428 |
| RP11-1336O20.2 | 4 | 5.68474913771228 | 0.00515038607051221 |
| RP11-192C21.2 | 4 | 2.24111984589884 | 0.0250183130037267 |
| RP11-161D15.2 | 4 | -2.504465444 | 0.0122636584366875 |
| RP11-169F17.1 | 18 | 3.51489963231306 | 0.0237760616445852 |
| AC007948.1 | 18 | 1.96764210057421 | 0.0491092320488915 |
| RP11-789C17.5 | 18 | -2.148681512 | 0.0316596552615628 |
| RP11-286N3.2 | 18 | 2.66334629091878 | 0.0335857944481645 |
| SNX19P3 | 18 | -2.425586707 | 0.0152836643189993 |
| RP11-94B19.5 | 18 | 3.68107180548617 | 0.000232255579506626 |
| RP11-775G23.1 | 18 | 6.02702509825827 | 0.00167402165805652 |
| OR4K7P | 18 | 1.97510590001301 | 0.0482561314344525 |
| RP11-94B19.2 | 18 | 2.14460182328358 | 0.0319846964077323 |
| RP11-100K18.1 | 18 | 5.8330885846175 | 0.00302919990796324 |
